# A combination of oral l-citrulline and l-arginine improved 10-min full-power cycling test performance in male collegiate soccer players: a randomized crossover trial

**DOI:** 10.1007/s00421-019-04097-7

**Published:** 2019-02-16

**Authors:** Izumi Suzuki, Keishoku Sakuraba, Takumi Horiike, Takafumi Kishi, Junya Yabe, Takashi Suzuki, Masahiko Morita, Akihito Nishimura, Yoshio Suzuki

**Affiliations:** 1 Faculty of Health and Sports Science, Juntendo University, 1-1, Hiragagakuendai, Inzai, Chiba 270-1695 Japan; 2 Research and Innovation Center, Kyowa Hakko Bio Co., Ltd, Miyukigaoka, Tsukuba, Ibaraki 305-0841 Japan

**Keywords:** Supplement, Pre-workout, Vasodilator, Ergogenic, Bicycle ergometry

## Abstract

**Purpose:**

Oral l-citrulline (Cit) increases plasma l-arginine (Arg) concentration and the production of nitric oxide (NO). NO dilates blood vessels and potentially improves sports performance. The combination of oral Arg and Cit (Arg + Cit) immediately and synergistically increases plasma Arg and nitrite/nitrate (NOx) concentrations more than either Cit or Arg alone. This prompted us to assess the effects of oral Arg + Cit on 10-min cycling performance in a double-blind, randomized, placebo-controlled crossover trial.

**Methods:**

Twenty-four male soccer players ingested either Cit + Arg or placebo (both 1.2 g/day each) for 6 days. On day 7, they ingested Cit + Arg 1 h before performing a 10-min full-power pedaling test on a bicycle ergometer. Plasma NOx and amino acid levels were measured before and after the test, as well as the participants’ subjective perception of physical exertion.

**Results:**

Power output was significantly greater with Cit + Arg than in the placebo group (242 ± 24 vs. 231 ± 21 W; *p* < 0.05). Plasma concentrations of post-exercise NOx (*p* < 0.05), Cit (*p* < 0.01) and Arg (*p* < 0.01) were significantly higher in the Cit + Arg than in the placebo group, whereas exercise upregulated plasma NOx concentrations in both groups (*p* < 0.05). Cit + Arg also gave improved post-exercise subjective perception of “leg muscle soreness” and “ease of pedaling” (both *p* < 0.05).

**Conclusion:**

Seven days of oral Citrulline (1.2 g/d) and Arginine (1.2 g/d) ingestion improved 10-min cycling performance and the perception of physical exertion in male collegiate soccer players.

## Introduction

Nitric oxide (NO) dilates blood vessels, enhancing circulation and improving mitochondrial efficiency (Albrecht et al. 2003; Tschakovsky and Joyner 2008; Larsen et al. 2011). A previous study suggests that NO positively regulates mitochondrial biogenesis through the upregulation of peroxisome proliferator-activated receptor-γ co-activator-1α (PGC-1α), a central regulator of mitochondrial function, because mitochondrial respiration and body energy balance are markedly abolished in eNOS-deficient animals (Nisoli et al. 2003). In addition, NO exerts a range of physiological functions [e.g., enhancing muscle contractile efficiency, improving exercise tolerance, and regulating oxygen consumption (Shen et al. 1994; Larsen et al. 2007; Bailey et al. 2010; Petróczi and Naughton 2010; Lansley et al. 2011a; Bescós et al. 2012; Jones et al. 2013)] acting as an intercellular messenger as well as forming reactive nitrogen species (Quijano et al. 2016). Some researchers have focused on the beneficial effects that dietary nitrate (NO_3_^−^)-containing supplements, such as beetroot juice, have on exercise performance (Bailey et al. 2009; Lansley et al. 2011b; Cermak et al. 2012a; Wilkerson et al. 2012; Jones 2014). However, other reports show no improvement in endurance performance after NO_3_^−^ supplementation in highly trained elite athletes (Cermak et al. 2012b; Boorsma et al. 2014). Therefore, efforts have been directed towards strategies that promote NO production.

The endogenous synthesis of NO proceeds via the metabolism of l-arginine (Arg) to l-citrulline (Cit) by NO synthase (Curis et al. 2005). Argininosuccinate synthase (ASS1) and argininosuccinate lyase (ASL) then recycle Cit to Arg, which results in a Cit–Arg cycle that efficiently produces NO (Curis et al. 2005).

Arg is a conditionally essential amino acid that exerts various physiological actions by improving vascular endothelial (Bode-Boger et al. 2003; Lin et al. 2008; Siasos et al. 2008), physical (Campbell et al. 2006; Fricke et al. 2008; Koppo et al. 2009), and sexual (Chen et al. 1999) functions. These functions require high doses of Arg, since intestine degrades substantial amount (~ 40%) of dietary Arg and the remainder is taken up by the liver and metabolized to urea (Castillo et al. 1993a, b; Wu 1998; Van De Poll et al. 2004).

Cit is an α-amino acid that is abundant in watermelon (*Citrullus vulgaris*) and is a potent endogenous precursor of Arg. Notably, oral Cit ingestion increases blood Arg concentrations more efficiently than an equal amount of Arg in humans (Schwedhelm et al. 2008). Cit is not metabolized in the small intestine or liver (Windmueller and Spaeth 1981; Van De Poll et al. 2007), which accounts for why oral Cit elevates Arg levels more effectively than Arg. We have recently demonstrated that Cit supplementation exhibits several beneficial effects on the cardiovascular system and endothelial function by enhancing NO production (Ochiai et al. 2012; Yabuki et al. 2013). A growing body of evidence also indicates that exercise performance is enhanced via NO production when healthy adults orally consume Cit (Bailey et al. 2015; Suzuki et al. 2016). Therefore, Cit could have physiological properties that efficiently elevate Arg and NO levels involving the modulation of exercise capacity.

Previous studies in animal models have shown that a combination of oral Cit and Arg increases blood Arg concentration immediately and more effectively than either Cit or Arg alone (Morita et al. 2014). Particularly in healthy humans, combined supplementation with Cit and Arg has been proven to exhibit acute elevation of plasma Arg levels within 1 h following intake: it then immediately demonstrates a half-life, t_1/2_, of 1.5–2 h (Suzuki et al. 2017). And in our previous study using the 4 km cycling time trial test 1 h after Cit supplementation (Suzuki et al. 2016), the mean completion time in Cit was significantly shorter than placebo (placebo: 578 ± 15 s, Cit: 569 ± 14 s, *p* < 0.05). Therefore, we hypothesized that a 10 min (600 s) full-power pedaling test at 1 h after intake, could detect the acute effects of oral Cit and Arg.

Previous studies (Bailey et al. 2015; Suzuki et al. 2016) have indicated that about 1 week of continuous Cit ingestion improves exercise performance, whereas a single bolus does not (Hickner et al. 2006; Cutrufello et al. 2015). The lowest dose and duration of Cit ingestion that is reportedly required to improve exercise performance is 2.4 g/day for 8 days (Suzuki et al. 2016). In addition, we found oral Cit and Arg combination synergistically increases plasma Arg levels compared with its ingestion of either alone (Morita et al. 2014; Suzuki et al. 2017). Therefore, we hypothesized that the Cit + Arg could exert the same effect at a lower dose of Cit. Here, we investigated the effects of 1.2 g/day each of oral Cit combined with Arg for 7 days on exercise performance.

## Materials and methods

### Participants

Twenty-four male soccer players aged 18 to 25 years volunteered to participate in this double-blind, randomized, placebo-controlled, two-arm crossover trial. All were members of Juntendo University’s Division 1 soccer team, which is part of the Kanto University Football League. The purpose, methods, potential results, and review of the trial protocol, as well as the protection of personal information, potential benefits, and disadvantages of participating in the trial were explained to each participant. All the players understood that participation depended on their own free will and that they could withdraw at any time. All players then provided written, informed consent to participate in the trial. The participants were habitually using the ergometer thereby expected to be familiarized enough to perform 10 min pedaling test. After the screening PWC test, they were instructed to practice the 10 min pedaling test at their prescribed load prior to the 1st trial so that to eliminate a learning effect.

The inclusion criteria were as follows: male collegiate soccer players, physical work capacity (PWC)_75%HRmax_ > 160 W, and ability to pedal a bicycle ergometer at > 60 rpm for 12 min with a workload set at their PWC_75%HRmax_ at 60 rpm.

The exclusion criteria were as follows: goal keepers, under medical treatment, presently with, or having a history of cardiovascular, respiratory endocrine or metabolic disorders, liver or kidney dysfunction, chest pain, fainting, allergies to components related to the test supplement, taking supplements that include Cit or Arg, smoking, and seasonal allergies such as *Cryptomeria japonica* or *Chamaecyparis obtusa* pollenosis, blood donations of > 200 mL, or of 400 mL within 1 or 3 months, respectively, before the bicycle ergometer test, participation in other clinical trials currently or within the past 3 months and being judged inappropriate by the study director.

The participants were instructed not to change their routine exercise habits and meal content during the study period.

The protocol was implemented according to the Declaration of Helsinki and was approved by the Ethics Committee of Juntendo University Graduate School of Sports and Health Sciences (Approval #27–108) as well as the Research Ethics Review Board of Kyowa Hakko Kirin Co., Ltd. (Approval #2015_043).

This study is registered at the UMIN clinical trial registry (UMIN-CTR; ID UMIN000021525).

Four participants were excluded from the analysis due to upper respiratory tract infections on the test day (*n* = 3) and missing data (*n* = 1). Data were analyzed from the remaining 20 participants (mean ± SE; age, 19.0 ± 0.2 years; weight, 65.4 ± 0.1 kg; height, 173.1 ± 1.1 cm; body mass index, 21.8 ± 0.2 kg/m^2^).

#### Physical work capacity tests

We measured PWC_75% HRmax_ as described by Miyashita et al. (Miyashita et al. 1985) using a PowerMax VIII bicycle ergometer, (Konami, Tokyo, Japan), with three stages of load (25, 75 and 125 W) for 3 min each (total, 9 min), and heart rate was measured using a heart rate monitor (RS 800 CX, Polar Japan, Tokyo, Japan). We calculated PWC_75% HRmax_ using a simple regression line derived from average heart rates for 30 s during the latter half of each stage and load intensity. Maximum heart rate was set at 220—age.

### Study design

The participants executed one trial with a placebo and another with Cit + Arg after a washout period of 2 months. The order of the trials was randomized so as a half of the participants to take Cit + Arg first and the others to take placebo first using research randomizer (https://www.randomizer.org/) in order to minimize the order effect.

The participants ingested the placebo or Cit + Arg for 7 days. Both were provided as granulated powders in a stick packet containing 2.4 g of maltitol (Mitsubishi Shoji Foodtech Co., Tokyo, Japan) or 1.2 g each of Cit and Arg (both Kyowa Hakko Bio Co., Ltd., Tokyo, Japan). Maltitol was used as a placebo because it has very similar appearance to Cit and Arg and no influences on our target outcomes to be evaluated. The appearance, weight, smell and taste of the Cit + Arg and placebo powders were confirmed to be indistinguishable by the manufacturer (Kyowa Hakko Bio Co., Ltd) of the test supplements and the practitioners prior to conduct this study.

Blood was collected from the cubital vein at the laboratory after an overnight fast on day 1, and then the supplement was ingested with 200 mL of water. Between days 2 and 6, Arg + Cit or the placebo were ingested before training or at bedtime on non-training days.

During days 1–6, the time of supplement ingestion and physical and health status were diarized. After 15:00 on day 6, the participants ingested only water, consumed a defined meal (1500 kcal, protein 49.1 g, lipid 63.1 g, carbohydrate 182.9 g) by 21:00 and then fasted.

The height, weight, blood pressure, heart rate, and self-reported physical and health status of the participants were evaluated at the laboratory, and blood was collected at 08:00 on day 7. The participants then consumed a rice ball (180 kcal, protein 3.6 g, lipid 0.5 g, carbohydrate 40.2 g) and water (*ad libitum* up to 500 ml) and rested for 60 min. The participants then ingested Cit + Arg or the placebo and rested for 35 min, warmed up for 15 min, rested for another 10 min (total of 60 min) and then started the 10-min pedaling test. This sequence was based on a report showing that plasma Arg levels peak at 1 h after supplementation (Suzuki et al. 2017).

The 10-min pedaling test was conducted using a PowerMax VIII bicycle ergometer (Konami, Tokyo, Japan), with the torque set to output individual PWC_75% HRmax_ at 60 rpm. Based on a previous study using the cycling time trial test (Suzuki et al. 2016), exercise conditions were set for evaluating full-power cycling test for about 10-min.

Blood was immediately collected after the 10-min pedaling test, then the participants provided responses to a visual analogue scale (VAS) questionnaire about their perception of physical exertion (muscle fatigue, leg muscle soreness) and subjective conditions (vigor, ease of pedaling, concentration, eyestrain, and blurred vision). The participants subjectively rated their degree of discomfort on a VAS from 0 (no discomfort) to 100 (extreme discomfort) mm after the 10-min pedaling test. The VAS was originally developed for measuring pain (Maxwell 1978) and has also been used to assess fatigue (Leung et al. 2004).

No adverse events developed during the study.

### Blood analysis

Plasma was prepared from blood samples by centrifugation at 1000×*g* and 4 °C for 10 min. Plasma (250 µL) was mixed with an equal volume of 6% trichloroacetic acid (for amino acid analysis) or 100% methanol (for NOx analysis), placed on ice for 1 h, separated by centrifugation at 13,000 × g and 4 °C for 10 min, and then the supernatant was separated. Free amino acids and NOx in deproteinized plasma were measured using an L-8900 amino acid analyzer (Hitachi High-Technologies Corporation, Tokyo, Japan) and an ENO-20 NOx analyzer (Eicom Corporation., Kyoto, Japan), respectively (Przyborowski et al. 2015).

### Statistical analysis

The number of participants was set to detect the difference in mean values by 0.60-fold of the standard deviation with an 80% power at an *α* level of 0.05 in the paired *t* test using R version 3.2.2. Individual power output was calculated as the mean power of the period, whereas the peak pedaling speed was the max pedaling speed of the period. Values are expressed as means or estimated marginal mean values ± standard error of the mean (SEM). The mean difference of the two paired groups was analyzed by paired t test if the normality was hypothesized by Shapiro–Wilk test, otherwise by Wilcoxon’s signed rank test. The mean difference of repeated measure data was analyzed by repeated measure ANOVA if the normality was hypothesized by Shapiro–Wilk test, otherwise by generalized linear model (generalized estimating equation procedure). Correlations were analyzed using Pearson’s correlation coefficients. All data was statistically analyzed using with SPSS Statistics 22 (IBM Japan, Ltd., Tokyo, Japan). *P* values < 0.05 and correlation coefficient ≥ 0.4 were regarded as significant.

## Results

### 10-min pedaling tests

**Fig. 1:**
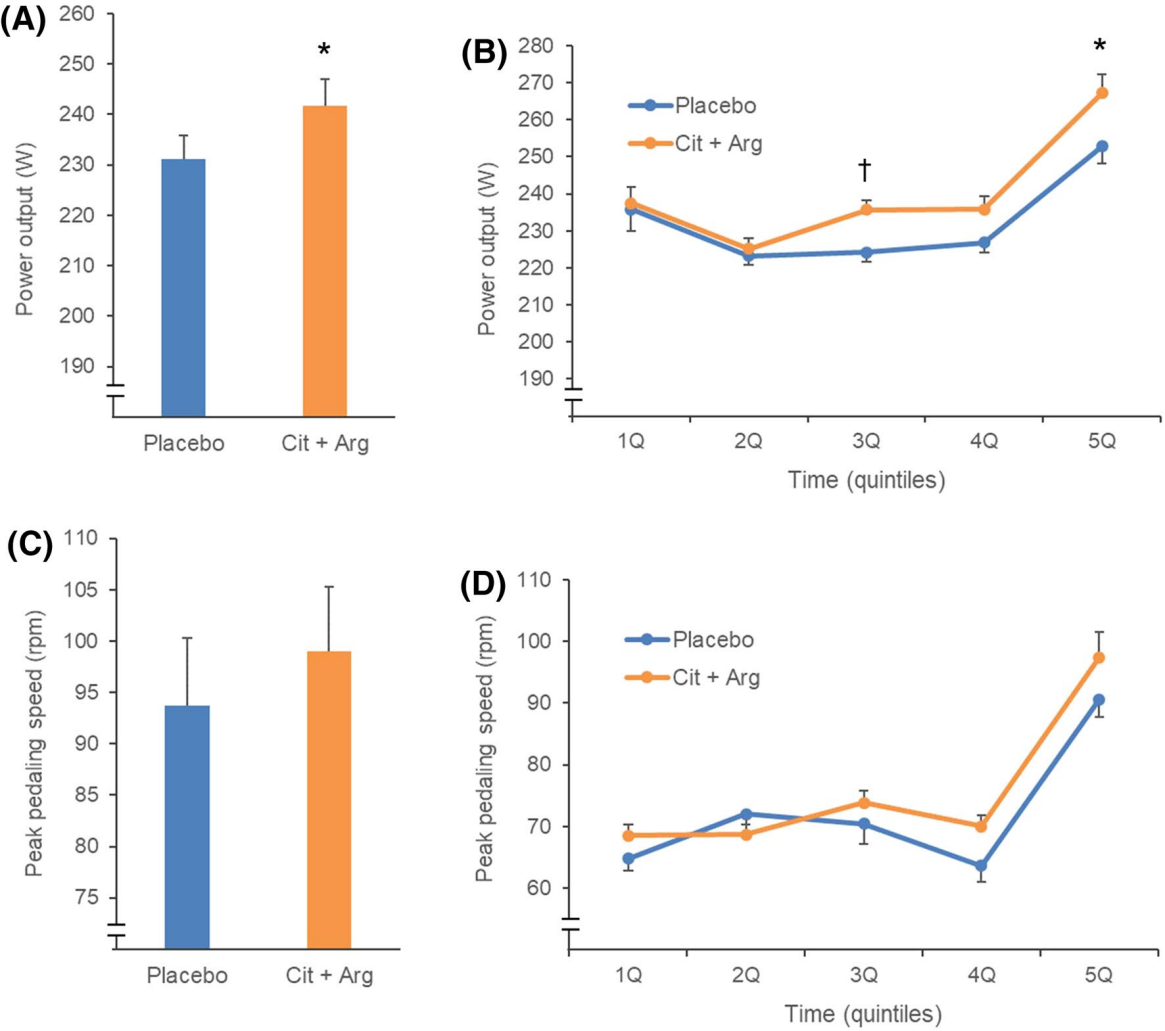
Results of 10-min pedaling exercise tests. Mean power output during total of 10 min (**a**) and the first to fifth quintiles (2 min each) (**b**). Max pedaling speed during total of 10 min (**c**) and the first to fifth quintiles (2 min each) (**d**). **a, c** were analyzed by paired *t* test, and **b** and **d** were analyzed by generalized estimating equation. Values are means ± SEM *n* = 20, *p < 0.05, ^†^*p* < 0.01 indicate a significant difference from placebo

Mean power output was significantly higher in the Cit + Arg group than in the placebo group throughout the 10-min pedaling test (242 ± 24 vs. 231 ± 21 W; *p* < 0.05; Fig. 1a) and in the third and fifth quintiles of the 10-min pedaling test (Fig. 1b).

The peak pedaling speed was not different between the Cit + Arg and placebo groups during the test (99.0 ± 28.4 vs. 93.8 ± 29.3 rpm; *p* = 0.297; Fig. 1c) nor in any quintile of the test (Fig. 1d).

### Plasma NOx on day 7

The 10-min pedaling test revealed significantly elevated plasma NOx concentrations in both groups (*p* < 0.05). However, the Cit + Arg group showed significantly higher plasma NOx concentration than that in placebo group after the test (45.2 ± 2.4 vs. 37.8 ± 2.4 µM; *p* < 0.05). The change in NOx concentrations before and after the exercise was significantly higher in the Cit + Arg group than in the placebo group (7.5 ± 1.3 vs. 3.7 ± 1.0 µM; *p* < 0.05).

### Plasma amino acids on day 7

**Fig. 2:**
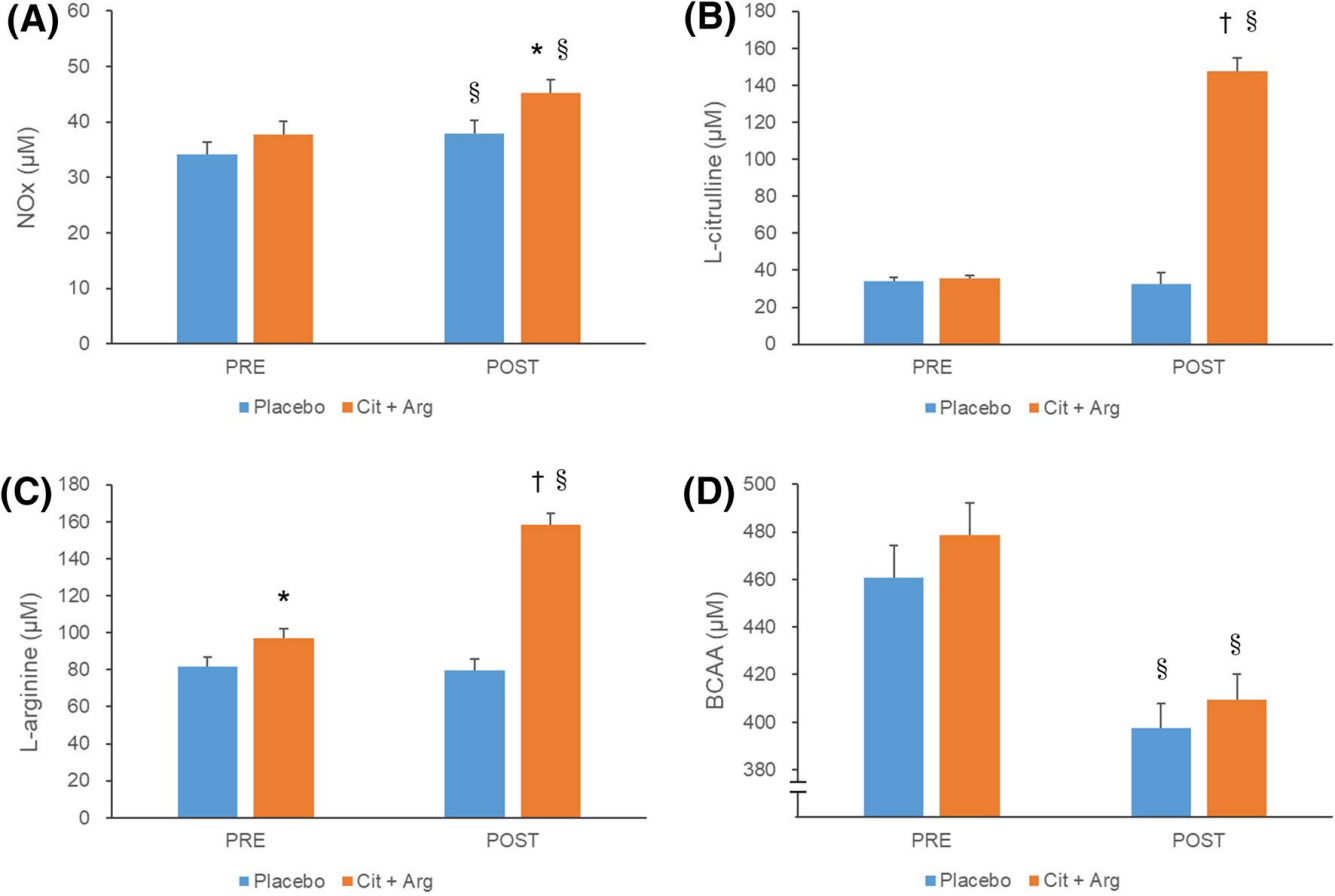
Plasma **a** NOx, **b**l-citrulline, **c**l-arginine, and **d** branched chain amino acids (BCAA) concentrations in placebo (blue bars) and Cit + Arg (orange bars) groups on day 7. Participants ingested Cit + Arg and then participated in 10-min ergometer cycle tests. Plasma NOx and amino acids were analyzed before supplement ingestion (PRE) and after exercise (POST). **a, c, d** Were analyzed by repeated measure ANOVA, and **b** was analyzed by generalized estimating equation. Height of bars and error bars represent mean values and SEM, respectively. Symbols above bars represent statistical significance as follows: *p < 0.05 and ^†^*p* < 0.01, between groups vs. placebo group, and ^§^*p* < 0.01, within groups vs. PRE

The plasma Arg level was significantly higher in the Cit + Arg than in the placebo group before the 10-min pedaling test (97.0 ± 5.0 vs. 81.7 ± 5.0 µM; *p* = 0.05; Fig. 2c). After the test, plasma Cit (147.4 ± 7.5 vs. 32.8 ± 5.8 µM; *p* < 0.01) and Arg (158.5 ± 6.1 vs. 79.8 ± 6.1 µM; *p* < 0.01) levels were significantly higher in the Cit + Arg group than in the placebo group. A comparison of plasma Cit and Arg levels before and after the exercise showed that plasma Cit levels did not change after the test in the placebo group, whereas plasma Cit (35.7 ± 1.4–147.4 ± 7.5 µM; *p* < 0.01) and Arg (96.9 ± 5.0–158.5 ± 6.1 µM; *p* < 0.01) levels were significantly higher in the Cit + Arg group post-exercise (Fig. 2b, c).

Levels of plasma branched chain amino acids (BCAA; l-valine, l-leucine, and l-isoleucine) were significantly lower after exercise, regardless of ingestion of Arg + Cit (478.7 ± 13.4 to 409.6 ± 10.4 µM; *p* < 0.01) or placebo (460.8 ± 13.4 to 397.4 ± 10.4 µM; *p* < 0.01) (Fig. 2d).

#### Subjective physical exertion and conditions

**Fig. 3:**
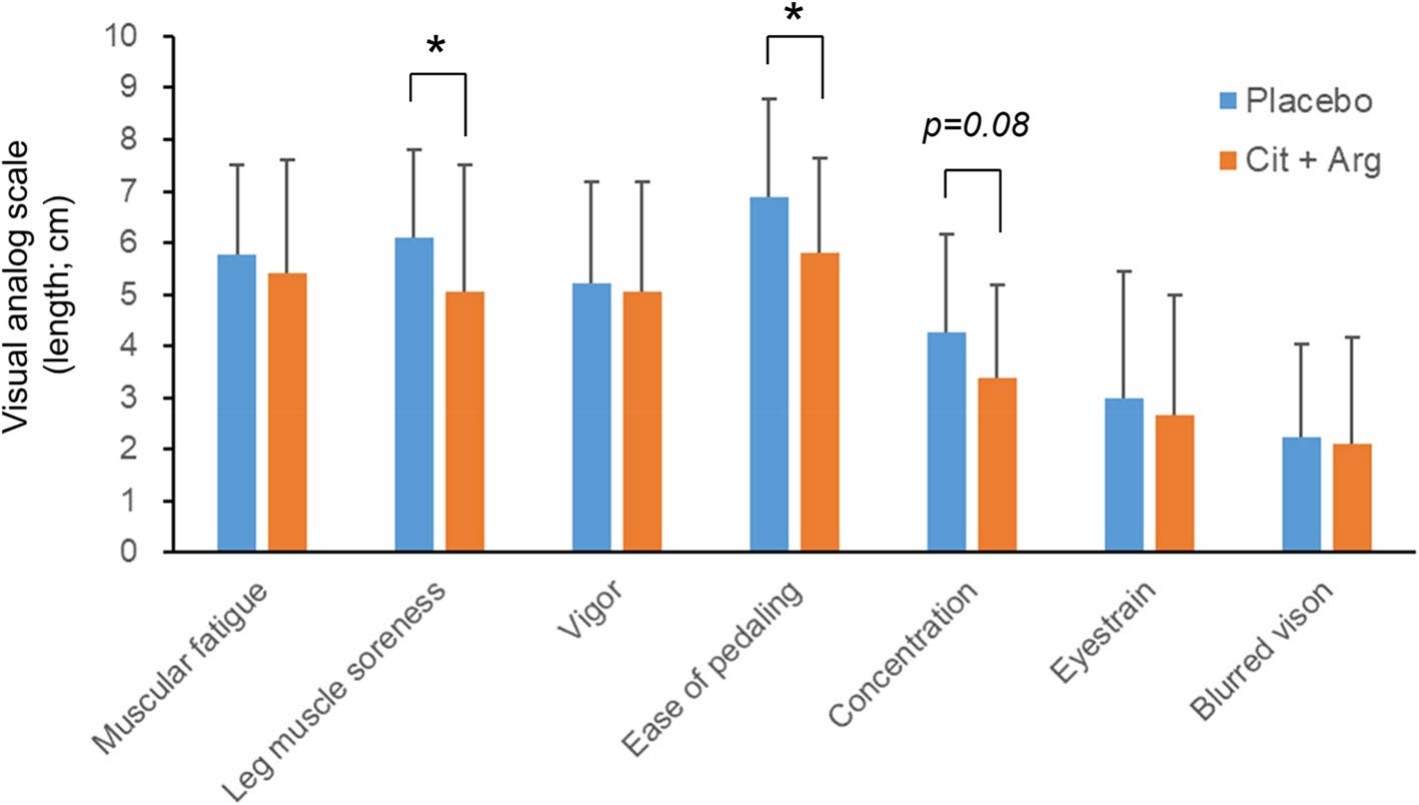
Subjective feelings immediately after exercise. Length represents perceived degree of discomfort on visual analog scale from 0 (no discomfort to 100 (extreme discomfort) mm, with higher values representing worsening discomfort. Mean difference was analyzed by Wilcoxon’s signed rank test, except for “Tension of the leg” and “Concentration” by paired *t* test. Values are means ± SEM, *n* = 20, *p < 0.05; significantly different from placebo

Supplementation with Cit + Arg significantly improved subjective perceptions of “leg muscle soreness” (5.1 ± 2.4 vs. 6.1 ± 1.7 cm; *p* < 0.05) and “ease of pedaling” (5.8 ± 1.8 vs. 6.9 ± 1.9; *p* < 0.05) when compared to the placebo immediately post-exercise. “Subjective concentration” (3.4 ± 1.8 vs.4.3 ± 1.9 cm; *p* = 0.08) also improved, whereas muscular fatigue and other subjective conditions did not differ between Cit + Arg and placebo (Fig. 3).

### Correlation between physical performance and NOx concentration

Power output during the final minute of physical exercise tended to correlate positively with plasma NOx concentration in the Cit + Arg group (*R* = 0.40, *p* = 0.08).

### Physical parameters

Height, weight, blood pressure (systolic and diastolic) and heart rate at rest did not significantly differ between the two groups before the exercise on day 7 (data not shown).

## Discussion

The ingestion of Cit + Arg (1.2 g/day each) for 7 days significantly increased 10 min cycling performance when compared to a placebo. In the Cit + Arg group, changes in plasma NOx levels before and after exercise and plasma Cit and Arg levels after exercise were significantly higher than in the placebo group. Immediately after the exercise, subjective perceptions of “leg muscle soreness” and “ease of pedaling” were also significantly improved in the Cit + Arg group.

Dietary NO_3_^−^-containing supplements, such as beetroot juice, have been reported to exert beneficial effects on exercise performance (Bailey et al. 2009; Lansley et al. 2011b; Cermak et al. 2012a; Wilkerson et al. 2012; Jones 2014). NO could be generated chemically: the ingested NO_3_^−^ enters the enterosalivary system and may subsequently be reduced to NO_2_ by bacterial activity, and NO_2_ could then be further reduced to NO (Duncan et al. 1995; Lundberg and Govoni 2004). On the other hand, plasma NO levels derived from Arg—Cit conversion (Schmidt et al. 1988) increase with exercise (Node et al. 1997), and are considered to enhance peripheral circulation and improve exercise performance (Shen et al. 1994; Larsen et al. 2007, 2011; Bailey et al. 2009; Lansley et al. 2011b; Cermak et al. 2012a; Wilkerson et al. 2012; Jones 2014). In fact, voluntary physical activities including running speed and distance are reduced in eNOS knockout mice (Momken et al. 2004). The present study found that Cit + Arg significantly increased plasma Cit, Arg and NOx levels in association with exercise when compared to a placebo. In addition, plasma NOx concentrations positively correlated with power output in the Cit + Arg group. Therefore, the elevated Cit and Arg values could enhance exercise-induced NO and improve cycling performance.

**Fig. 4:**
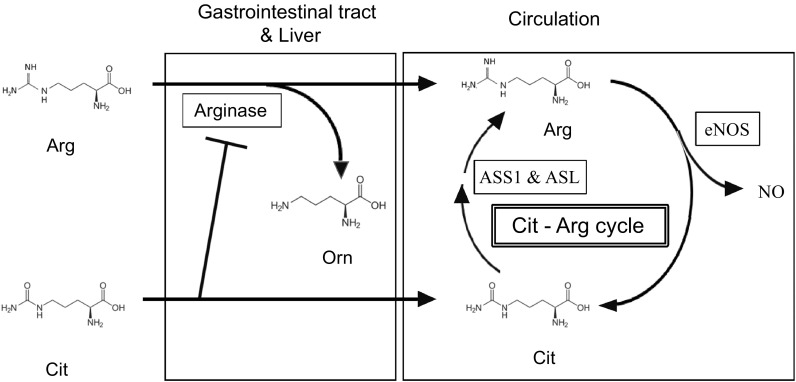
Metabolic pathways of l-arginine (Arg) and l-citrulline (Cit). Ingested Arg is mainly degraded in gastrointestinal tract and liver by arginase which is inhibited by Cit. Therefore, co-ingested Cit protects Arg and elevates circulating Arg concentrations. Endothelial nitric oxide (NO) synthase (eNOS) converts Arg to NO and Cit in circulation. Thereafter, Cit is then recycled to Arg by argininosuccinate synthase (ASS1) and argininosuccinate lyase (ASL). This Cit–Arg cycle enables effective NO production

Ingested Arg is susceptible to arginase degradation to ornithine in the gastrointestinal tract and liver (Fig. 4) and thus it cannot effectively elevate or maintain plasma levels of Arg. We previously showed that plasma Arg levels returned to the baseline within 4 h after the ingestion of 2.0 g of Arg (Suzuki et al. 2017). This could account for why high doses of 6–14.2 g/day are needed to improve exercise function in healthy persons (Campbell et al. 2006; Fricke et al. 2008; Koppo et al. 2009).

Cit is not metabolized in the small intestine, and it inhibits the degradation of Arg by arginase. Thus, oral Cit + Arg synergistically increases plasma Arg levels in mice and humans compared with its ingestion of either alone (Morita et al. 2014; Suzuki et al. 2017). Circulating Arg is converted to NO and Cit by eNOS. Cit then is recycled to Arg by ASS1 and ASL in an efficient cycle of NO production in the circulatory system (Fig. 4) (Curis et al. 2005). In this study, plasma Cit levels increased significantly after Cit + Arg intake. This may provide an explanation, in that arginase suppression, mediated by Cit, probably contributes to a marked increase in Arg availability.

However, our previous study did not show elevated Arg levels on the day after ingesting Cit and Arg (1.0 g/day of each) for 7 days (Suzuki et al. 2017). The present study found that orally ingested Cit + Arg (1.2 g/day each for 6 days) significantly increased plasma Arg levels when compared to a placebo before Cit + Arg ingestion on day 7. The participants did not ingest the supplement after 15:00 on day 6, and blood was collected > 17 h after the final ingestion. The combination of Cit + Arg (1.2 g/day each) therefore increased and maintained plasma Arg concentrations.

The discrepancy in plasma Arg concentrations between the previous (Suzuki et al. 2017) and the present studies could be due to differences in the doses and the background characteristics of the participants, who consisted of obese men in the previous study (Suzuki et al. 2017) and collegiate athletes in the present study. Arginase activity is higher in patients with hypertension and diabetes, and this decreases Arg bioavailability (Shatanawi et al. 2011; Pernow and Jung 2013). Another study has shown that the ingestion of 2.4 g/day of Cit for 7 days increased plasma Arg levels on day 8 in men who regularly exercised (Suzuki et al. 2016). Another possible explanation for the immediate and synergistic increase in plasma Arg and NO by oral Cit + Arg ingestion might be the inhibition of arginase by Cit (Romero et al. 2008). Cit suppresses arginase activity *in vitro* and *in vivo* through its powerful allosteric inhibitory action (Shearer et al. 1997; Romero et al. 2008; El-Bassossy et al. 2012). The Arg transporters rBAT/b0, AT, and CAT-1 and the Cit transporter SN-1 are simultaneously expressed in intestinal epithelial and vascular endothelial cells (Closs et al. 2004; Broer 2008; Romero et al. 2008). Thus, Cit might be absorbed together with Arg when combined Cit and Arg are ingested, inhibiting arginase in intestinal and endothelial cells, and thus increasing Arg concentrations and NO production. The elevated plasma Cit concentration in the present study could account for this mechanism to increase plasma Arg and NO concentration. Therefore, Cit + Arg, at relatively low dosages, could increase Arg levels, especially in athletes.

Some studies have shown that a single administration of oral Cit does not improve exercise performance (Hickner et al. 2006; Cutrufello et al. 2015), whereas 6–7 days of Cit supplementation improves exercise tolerance (Bailey et al. 2015; Suzuki et al. 2016). The present study showed performance in the 10-min pedaling test to be enhanced after 7 days of Cit + Arg supplementation. These findings suggest that, to improve exercise performance, Cit and Arg should be continuously ingested. Arginine and Cit, both individually and in combination, elevate endothelial NO synthase levels and decrease those of arginase in human vascular endothelial cells (HUVECs) after 3 days of incubation (Tsuboi et al. 2018). The duration of Arg and Cit ingestion might have altered the endothelial capacity for NO production.

Other factors require further exploration. Two reports have indicated that the oral intake of Cit significantly decreases plasma BCAA levels (Sureda et al. 2010; Suzuki et al. 2016), but the present study found no significant differences in these levels. The relationship between Cit and BCAA remains to be determined. l-citrulline-malate and watermelon juice, which is rich in Cit, can alleviate the delayed onset of muscle soreness (Pérez-Guisado and Jakeman 2010; Tarazona-Díaz et al. 2013). The present study did not measure muscle pain on the day after the exercise test or beyond, but subjective perceptions of “leg muscle soreness” and “ease of pedaling” immediately after the exercise were improved. Therefore, Cit or the resulting NO could influence subjective perceptions, and the mechanism behind this requires further investigation.

The present study had several limitations. The kinetics of plasma amino acids and NOx concentrations were not measured. Notably, increases in plasma NOx levels induced by the exercise were much lower than those measured in the previous literature (Jungersten et al. 1997; Franco et al. 2001; Suzuki et al. 2016). The discrepancy could be due to the timing of blood collection. We collected post-exercise blood immediately after the exercise; nevertheless, the NOx concentration induced by high-intensity exercise is reported to be elevated for up to 24 h after exercise (Güzel et al. 2007), which could suggest that the time-point for collecting the blood in our study was not suitable for detecting the peak in nitrate levels. Because a significant difference was observed in post-exercise NOx concentrations, the difference could have been more apparent had we checked later, preferably up to 48 h after the exercise. Therefore, a closer study of the timing of supplementation and exercise is needed. This study indicated 1.2 g/day of Cit is the minimum dose needed to evaluate a potential beneficial effect on exercise performance, but that this is combined with the same amount of Arg. The dose–response effect of Cit + Arg needs to be more precisely determined. The duration of the supplementation should also be assessed, because we tested a duration of 7 days in vivo, whereas NO synthase and arginase levels are up-regulated in HUVECs in vitro within 3 days (Tsuboi et al. 2018). Furthermore, our participants comprised 20 male collegiate soccer players. Thus, more participants with various backgrounds, including females, sedentary persons and recreational athletes, should be evaluated to give the conclusion a broader relevance.

## Conclusion

Oral ingestion of Cit and Arg at doses of 1.2 g/day each for 7 days improved exercise performance in a 10-min pedaling test and the participants’ subjective perceptions of physical exertion. The increased NO production induced by elevated plasma Cit and Arg levels could account for this effect.

## Abbreviations

Arg: l-arginine
ASL: Argininosuccinate lyase
ASS1: Argininosuccinate synthase
BCAA: Branched chain amino acids
Cit: l-citrulline
NO: Nitric oxide
NOx: Nitrite/nitrate
NO_2_: Nitrite
NO_3_^−^: Nitrate
SEM: Standard error of the mean
VAS: Visual analogue scale
